# Characterization of the complete mitochondrial genome and phylogenetic analysis of *Leucauge celebesiana* (Araneae: Tetragnathidae)

**DOI:** 10.1080/23802359.2019.1673248

**Published:** 2019-10-03

**Authors:** Xin Yan, Kang-Kang Xu, Da-Xing Yang, Can Li, Wen-Jia Yang

**Affiliations:** Guizhou Provincial Key Laboratory for Rare Animal and Economic Insect of the Mountainous Region, College of Biology and Environmental Engineering, Guiyang University, Guiyang, Guizhou, China

**Keywords:** *Leucauge celebesiana*, Tetragnathidae, mitogenome

## Abstract

The complete mitogenome of *Leucauge celebesiana* (GenBank accession number MN296353) is 13,901 bp in size, and harbours 13 protein-coding genes (PCGs), 22 transfer RNAs (tRNAs), 2 ribosomal RNAs, and an A + T-rich region. The base composition of the mitogenome comprised A (33.80%), C (8.82%), G (14.42%), and T (42.96%), with a total A + T content of 76.76%. Eleven tRNAs (*trnK*, *trnP*, *trnL_1_*, *trnD*, *trnF*, *trnG*, *trnH*, *trnR, trnT*, *trnW*, and *trnV*) lacked the TΨC arm stem, while three tRNAs (*trnA*, *trnS_1_*, and *trnS_2_*) lost the dihydrouracil (DHU) arm. Phylogenetic analysis suggests that *L. celebesiana* has a close phylogenetic relationship with *Tetragnatha maxillosa*, which agree with the traditional taxonomy.

The orb-weaving spider, *Leucauge celebesiana* (Araneae: Tetragnathidae), is an important predator of many agricultural pests (Nasir et al. 2016). This species is widely distributed in China, India, Japan, Indonesia, and New Guinea, and usually lives in paddy fields, waterside plants and shurblands (Li et al. 2011). In this study, adult individuals of *L. celebesiana* were collected from the Maolan Nature Reserve in Libo county, Guizhou Province, China (N25°18′, E107°52′). Samples were preserved in 95% ethanol and stored in the spider specimen room of Guiyang University with an accession number GYU-GZML-18.

The complete mitochondrial genome of *L. celebesiana* (GenBank accession number MN296353) is 13,901 bp in size, harbouring 37 typical animal mitochondrial genes which include 13 protein-coding genes (PCGs), 22 transfer RNAs (tRNAs), 2 ribosomal RNAs (*rrnL* and *rrnS*), and an A + T-rich region (Boore 1999). The gene content and orientation of *L. celebesiana* were consistent with those observed in other spider mitogenomes (Wang et al. 2016; Xu et al. 2019). Sixteen genes were transcribed on the minority strand (N-strand), while the others were encoded on the majority strand (J-strand). The overall base composition of *L. celebesiana* mitogenome comprised A (33.80%), C (8.82%), G (14.42%), and T (42.96%), with a total of A + T content of 76.76%. The AT-skew and GC-skew of this genome were −0.119 and 0.241, respectively. A total of 208 bp overlaps were found at 27 gene junctions in *L. celebesiana* mitogenome, and the length of overlaps are ranging from 1 to 28 bp. Intergenic spacers were present in five positions and involved a total of 19 bp, the longest intergenic spacer was located between *trnF* and *nad5*.

The length of 22 tRNAs ranged from 51 bp (*trnS_1_* and *trnV*) to 118 bp (*trnC*), A + T content ranged from 66.13% (*trnM*) to 88.14% (*trnC*). Fourteen tRNAs lacked the potential to form the cloverleaf secondary structure. Eleven tRNAs (*trnK*, *trnP*, *trnL_1_*, *trnD*, *trnF*, *trnG*, *trnH*, *trnR, trnT*, *trnW*, and *trnV*) lacked the TΨC arm stem, while three tRNAs (*trnA*, *trnS_1_*, and *trnS_2_*) lost the dihydrouracil (DHU) arm. The *rrnL* was 1,026 bp long with A + T content of 80.60%, and the *rrnS* was 694 bp long with A + T content of 82.71%. The *rrnL* was located between *trnL_1_* and *trnV*, and *rrnS* resided between *trnV* and *trnQ*. The A + T-rich region was located between *trnQ* and *trnM* genes with a length of 377 bp long and the A + T content was 83.29%.

Nine PCGs start with typical ATN codons (ATT for *nad1*, *nad2*, *nad4L*, and *atp8*; ATA for *atp6*, *nad3*, *nad4*, and *nad5*; ATG for *cob*). However, three genes (*cox2*, *cox3*, and *nad6*) start with TTG, and *cox1* uses TTA as initiation codon. Ten PCGs terminate with conventional stop codons (TAA or TAG), and the remaining PCGs including *nad5*, *nad4L*, and *cob* use a single T as termination codon. According to the relative synonymous codon usage analyses of 13 PCGs, ATA (M), TTT (F), ATT (I), TTA (L) and were the four most frequently used codons. Methionine, phenylalanine, isoleucine, and leucine are the most frequent the amino acid of 13 PCGs. Based on the concatenated amino acid sequences of 13 PCGs, the neighbour-joining method was used to construct the phylogenetic relationship of *L. celebesiana* with 16 other representative spiders. The result showed that *L. celebesiana* is closely related to *Tetragnatha maxillosa* (Figure 1), which agree with the traditional taxonomy.

**Figure 1.**
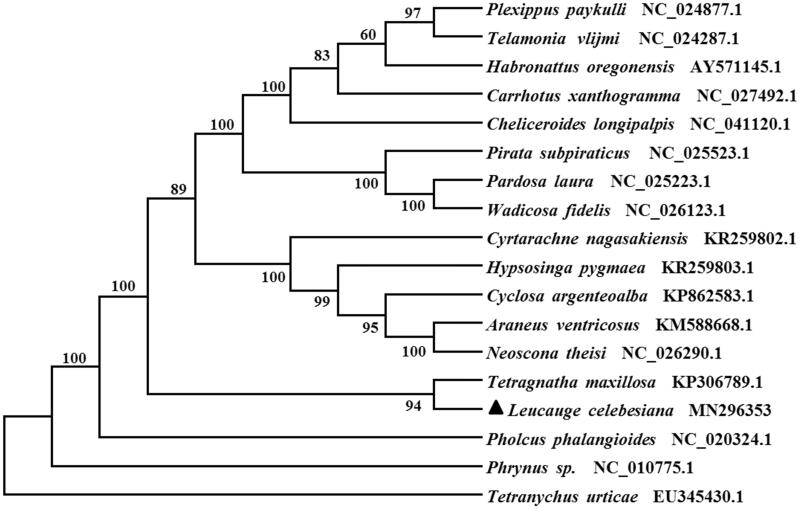
Phylogenetic tree showing the relationship between *Leucauge celebesiana* and 16 other representative spiders based on neighbour-joining method. GenBank accession numbers of each species were listed in the tree. Spider determined in this study is labelled with triangle. *Tetranychus urticae* was used as an outgroup.

## References

[CIT0001] Boore JL 1999 Survey and summary: animal mitochondrial genomes. Nucleic Acids Res. 27:1767–1780. 10101183 10.1093/nar/27.8.1767 PMC148383

[CIT0002] Li Z, Wang LY, Li ZX, Zhang ZS 2011 Analysis on community diversity of spiders from Chishui national nature reserve for spinulose tree fern, Guizhou province. Sichuan J Zool. 30:972–976.

[CIT0003] Nasir DM, Xing WC, Bakri A, Rahim F, Yusoff NR 2016 Spiders of Sabah: fifty new records including the description of a new *Leucauge* species. Biodiversitas. 17:799–807.

[CIT0004] Wang ZL, Li C, Fang WY, Yu XP 2016 Characterization of the complete mitogenomes of two *Neoscona* spiders (Araneae: Araneidae) and its phylogenetic implications. Gene. 590:298–306. 27259661 10.1016/j.gene.2016.05.037

[CIT0005] Xu KK, Lin XC, Yang DX, Yang WJ, Li C 2019 Characterization of the complete mitochondrial genome sequence of *Neoscona scylla* and phylogenetic analysis. Mitochondrial DNA Part B. 4:416–417.

